# Non-linear ICA Analysis of Resting-State fMRI in Mild Cognitive Impairment

**DOI:** 10.3389/fnins.2018.00413

**Published:** 2018-06-19

**Authors:** Xia-an Bi, Qi Sun, Junxia Zhao, Qian Xu, Liqin Wang

**Affiliations:** College of Information Science and Engineering, Hunan Normal University, Changsha, China

**Keywords:** resting state networks, mild cognitive impairment, functional magnetic resonance imaging, functional connectivity, post-non-linear independent component analysis

## Abstract

Compared to linear independent component analysis (ICA), non-linear ICA is more suitable for the decomposition of mixed components. Existing studies of functional magnetic resonance imaging (fMRI) data by using linear ICA assume that the brain's mixed signals, which are caused by the activity of brain, are formed through the linear combination of source signals. But the application of the non-linear combination of source signals is more suitable for the mixed signals of brain. For this reason, we investigated statistical differences in resting state networks (RSNs) on 32 healthy controls (HC) and 38 mild cognitive impairment (MCI) patients using post-nonlinear ICA. Post-nonlinear ICA is one of the non-linear ICA methods. Firstly, the fMRI data of all subjects was preprocessed. The second step was to extract independent components (ICs) of fMRI data of all subjects. In the third step, we calculated the correlation coefficient between ICs and RSN templates, and selected ICs of the largest spatial correlation coefficient. The ICs represent the corresponding RSNs. After finding out the eight RSNs of MCI group and HC group, one sample *t*-tests were performed. Finally, in order to compare the differences of RSNs between MCI and HC groups, the two-sample *t*-tests were carried out. We found that the functional connectivity (FC) of RSNs in MCI patients was abnormal. Compared with HC, MCI patients showed the increased and decreased FC in default mode network (DMN), central executive network (CEN), dorsal attention network (DAN), somato-motor network (SMN), visual network(VN), MCI patients displayed the specifically decreased FC in auditory network (AN), self-referential network (SRN). The FC of core network (CN) did not reveal significant group difference. The results indicate that the abnormal FC in RSNs is selective in MCI patients.

## Introduction

Independent component analysis (ICA) is a popular blind source separation technique and a powerful data-driven method (Dipasquale et al., 2015). ICA is able to decompose complex magnetic resonance signal patterns, and detect the resting state networks (RSNs) from functional magnetic resonance imaging (fMRI) data of all subjects (Abou Elseoud et al., 2011). The advantage of ICA is that it does not require prior information when extracting brain maps and time courses from fMRI data (Svensén et al., 2002). By contrast, other analysis methods of fMRI data require prior information, and the prior information is generally artificial which may lead to the error of the result. For example, the selection of regions of interest (ROIs) in seed correlation analysis method is artificially set (Koenig et al., 2009). As ICA does not require prior information, this data-driven method is widely used in the analysis of fMRI data (Robinson and Schöpf, 2013).

In general, ICA is divided into temporal ICA and spatial ICA (Mckeown et al., 1998). As ICA can separate independent components (ICs) in any order, two strategies are ordinarily used in studies to compare ICs of different subjects (Calhoun et al., 2009). In the first strategy, ICA is respectively performed on each subject's data, and then the relationship between ICs of each subject is established by the means of subjective identification (Calhoun et al., 2001a), and clustering (De Martino et al., 2007). In the second strategy, ICA is performed on the group data, then a subject's specific ICs are acquired from group ICs and the relationship between ICs of different group is also established. The second strategy is called group ICA (Calhoun et al., 2009).

At present, the fMRI studies using linear ICA could be divided into two streams. One stream is the application of linear ICA to fMRI based on the task state such as complex tasks (Kohler et al., 2008) and multimodal stimuli (Malinen et al., 2007). For example, van de Ven et al. (2008) analyzed the fMRI data of 9 subjects by employing the self-organizing group ICA (sogICA), and the fMRI data of subjects was collected through a three-stimulus visual oddball task (van de Ven et al., 2008). On the other hand, Malinen et al. (2007) used ICA and general-linear-model-based analysis (GLM) to analyze the fMRI data of 6 subjects, and the results showed that ICA was found to be a sensitive tool for studying brain responses to complex natural stimuli compared with GLM (Malinen et al., 2007).

The other stream is the application of linear ICA to fMRI based on the resting state (Li S et al., 2016). Esposito et al. (2008) used linear ICA to study the relationship between age and the default mode (DM) regions activity. They studied the effects of aging on DM components by employing resting state fMRI data from 20 healthy subjects. By combining the results of individual ICA and group ICA, they found that the DM connectivity was negatively correlated with age (Esposito et al., 2008).

Existing studies of fMRI data based on task state or resting state by using ICA assume that the brain's mixed signals, which are caused by the activity of brain, are formed through the linear combination of source signals (Du et al., 2011). In fact, the brain is made up of about 10^11^ nerve cellsand and connected by the 10^15^ nerve synapses, which is one of the most complex systems in the universe (Poldrack and Farah, 2015). Because the brain is so complex, most brain function activities are featured with nonlinearity. Therefore, linear ICA is not the best way to explore the mechanism of the abnormal functional connectivity (FC) of brain in patients. Non-linear ICA can be divided into many types, but not all of them are suitable for studying the abnormal FC of brain in patients. In this study, we adopt the post-nonlinear ICA (Szabo et al., 2007) with the motivation of combining the linear part of brain activity and the non-linear part of brain activity. The linear and non-linear brain activities help us study the abnormal FC in the scenario which is more in line with actual situation. In the post-nonlinear ICA, the signal firstly goes through a linear channel, and then is introduced by non-linear characteristics, and finally the mixed signal is formed (Wei et al., 2018). We apply this method to explore the RSNs of MCI and healthy controls (HC), and abnormal RSNs of MCI patients are found out by comparing the RSNs of HC and MCI patients. This method provides a novel way for diagnosis of MCI.

## Materials and methods

### Subjects

The experimental data of this study comes from the open database Alzheimer's Disease Neuroimaging Initiative (ADNI) (http://adni.loni.ucla.edu/). This database was established by a number of non-profit organizations in 2003, and provides comprehensive data including structural MRI data, functional MRI data and positron emission tomography data of Alzheimer's disease (AD), MCI and some healthy elderly. The patients' detailed basic information and clinical information are also provided in the database. This study used the functional MRI data of MCI patients and healthy elderly in this database. 71 subjects including 38 MCI patients and 33 HC were obtained. The subjects were excluded if the translation exceeded ±2.5 mm and rotation exceeded ±2.5. Finally, the remaining 70 subjects were involved in this study, including 38 MCI patients (age: 72.99 ± 7.79; 23 m/15 f) and 32 HC (age: 76.25 ± 6.51; 13 m/19 f).

There was no difference (*P* = 0.097) in gender between the two groups by chi-square test. We conducted two-sample *t*-tests on the age of MCI patients and HC, and found no difference (*P* = 0.64). Clinical diagnosis of MCI was confirmed with the mini mental state examination (MMSE) and the clinical dementia rating scale (CDR). The demographic information for MCI group and HC group is listed in Table 1.

**Table 1:** Demographic information table of all subjects.

**Project**	**MCI (*n* = 38)**	**HC (*n* = 32)**	***P* value**
Gender (Male/Female)	23/15	13/19	0.097^a^
Age	72.99 ± 7.79	76.25 ± 6.51	0.064^b^
MMSE	27.11 ± 2.44	29.13 ± 1.31	0.000^b^
CDR	0.54 ± 0.14	0.00 ± 0.00	–

a *The p-value is obtained through the chi-square test*.

b *The p-value is obtained by the two-sample t-tests, and the data in the table is represented by the mean ± standard deviation*.

### Image acquisition

Functional MRI data of MCI patients and HC was collected on the 3.0 T-MRI scans of Philips medical system. All subjects were ordered to remain quiet, lie flat in the scanner, and try to stay still and not think about any problem. The detailed description of the sequence parameters related with functional images of all subjects is as follows: TR = 3,000 ms, TE = 30 ms, flip angle = 80, matrix = 64 × 64, Pixel Spacing = 3.3 × 3.3, 3 mm thickness, without gap, number of volumes = 130, 48 slices.

### Data preprocessing

DPARSF software has helped us complete the data preprocessing (http://d.rnet.co/DPABI/DPABI_V2.3_170105.zip). The description of data preprocessing is as follows:

The original data collected from the database was in DICOM format which could not be recognized by the preprocessing software DPARSF. Thus, the original data was firstly converted to the NIFTI format. Secondly, as the scanner needs a certain amount of time to achieve a stable state, the first ten volumes were discarded to make the scanner stable. Thirdly, the remaining 120 volumes of each subject were corrected for the temporal difference in order to ensure that the data was collected at the same time. The head motion correction excluded one of the 33 HC, because the subject's translation exceeded ±2.5 mm and rotation exceeded ±2.5. Then the spatial normalization was performed by using EPI templates to eliminate differences in individual brains. Subsequently, the data was smoothed by Gaussian kernel (*FWHM* = 6 mm). In existing studies, the final step of data preprocessing for ICA is Gaussian smoothing (Liao et al., 2010a). As the subsequent calculation of the FC based on voxels was required (Wei et al., 2017b), the data preprocessing in our paper also included the linear drift and filter (0.01~0.08 HZ).

### Determination of RSNs

This paper used the post-nonlinear ICA method^1^ to extract ICs of MCI patients and ICs of HC by using GIFT software (http://icatb.sourceforge.net/) (Liao et al., 2010a). We firstly used the “Minimum description length” criterion (Jafri et al., 2008) provided by the GIFT software to estimate the number of ICs of the two groups. The estimated number of ICs in MCI patients and HC was 30 and 29, respectively. Then principal component analysis was performed in order to reduce the temporal dimension of fMRI data for all subjects. Finally, ICs were estimated by fast ICA algorithm based on post-nonlinear ICA. The post-nonlinear ICA was carried out separately in MCI group and HC group. Subsequently, the 30 ICs in MCI group and the 29 ICs in HC group were obtained. These ICs include time-course and spatial maps.

The time-courses of ICs reflect the waveform of brain activity, and spatial maps of ICs reflect brain activity intensity of voxel. To show the voxel which makes the largest contribution to a specific IC, we converted the intensity values of spatial map to *Z*-values (Calhoun et al., 2001b; Mantini et al., 2007). Z-value is generally considered to be the most effective way to measure the FC of intrinsic network (Bartels and Zeki, 2005; Damoiseaux et al., 2006). After obtaining ICs of two groups, we used Gift software to calculate the spatial correlation coefficients between eight RSNs templates and ICs, and selected the IC of the largest spatial correlation coefficient (Greicius et al., 2007; Wu et al., 2017a). The selected IC represents its corresponding RSN, and is retained for subsequent studies. The eight RSNs templates are provided by Dante Mantini from Leuven Medical School (Mantini et al., 2007, 2009). The eight RSNs templates are respectively DMN, DMN, CEN, VN,AN, SRN, SMN, DAN,CN.

### Two analysis methods for RSNs

After finding out the eight RSNs of MCI group and HC group according to the largest spatial correlation principle (Greicius et al., 2007), the spatial maps corresponding to each RSNs of the two groups were collected to perform one-sample *t*-tests. The results of one-sample *t*-tests were presented at the given threshold of *T* > 2. One-sample *t*-tests help to find out the activated brain regions, but they could not be used to test the significance of differences in RSNs. We further used two-sample *t*-tests to compare the differences in the FC of eight RSNs. The null hypothesis of two-sample *t*-tests is that there are differences of the FC of RSNs in MCI group and HC group. Before performing two-sample *t*-tests, a union of the results of one-sample *t*-tests of MCI group and HC group was firstly formed. Then the FC based on the voxels of 70 subjects was calculated by regarding the union as the regions of interest (ROIs). Thirdly, Z-transform of the FC was performed. Finally, the two-sample *t*-tests were carried out, and the results were displayed at the given threshold of *P* < 0.05 (AlphaSim correction).

## Results

### Spatial pattern of RSNs in each group

**Figure 1:**
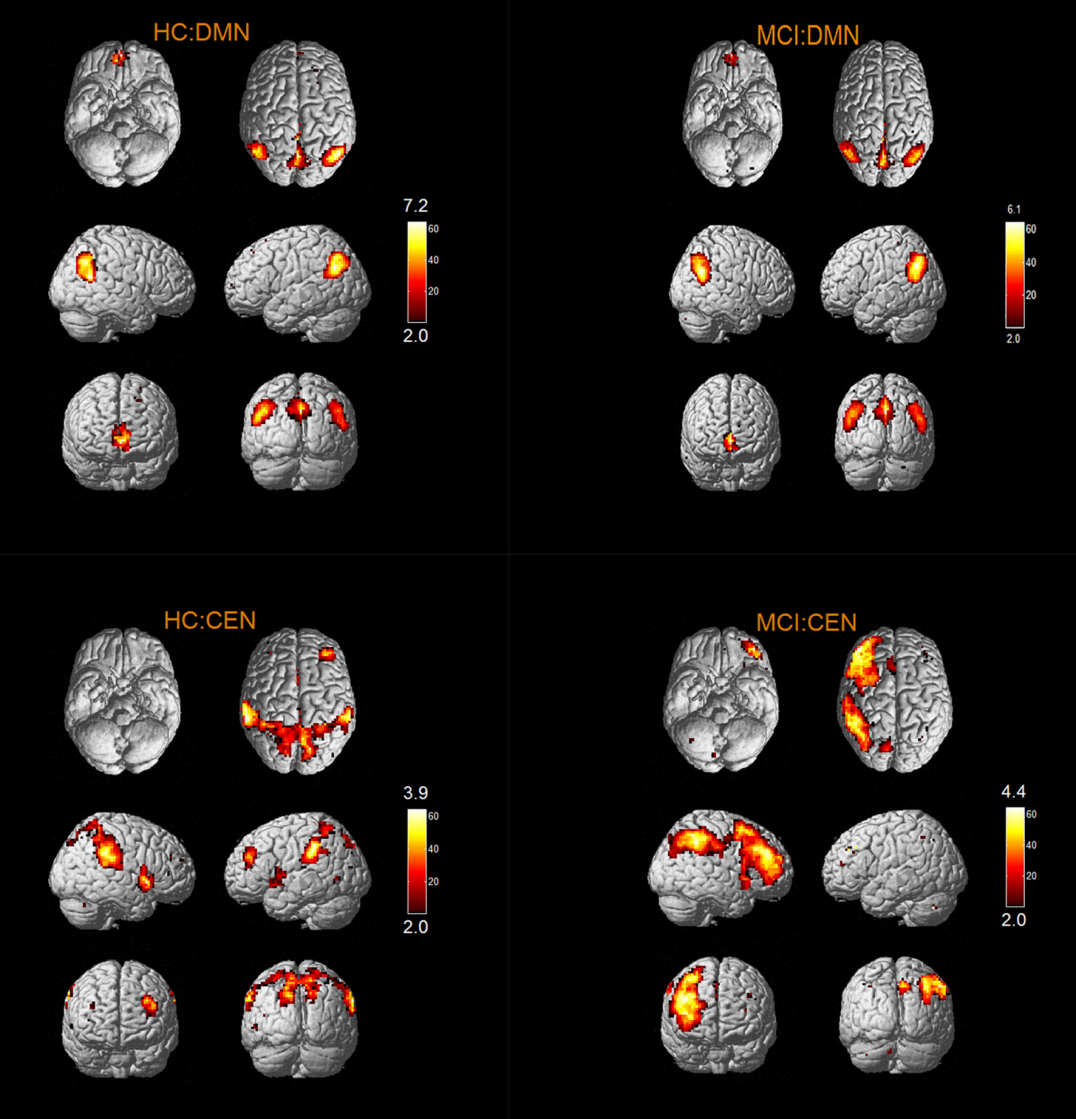
The spatial distribution of DMN, CEN, SMN in MCI group and HC group.

**Figure 2:**
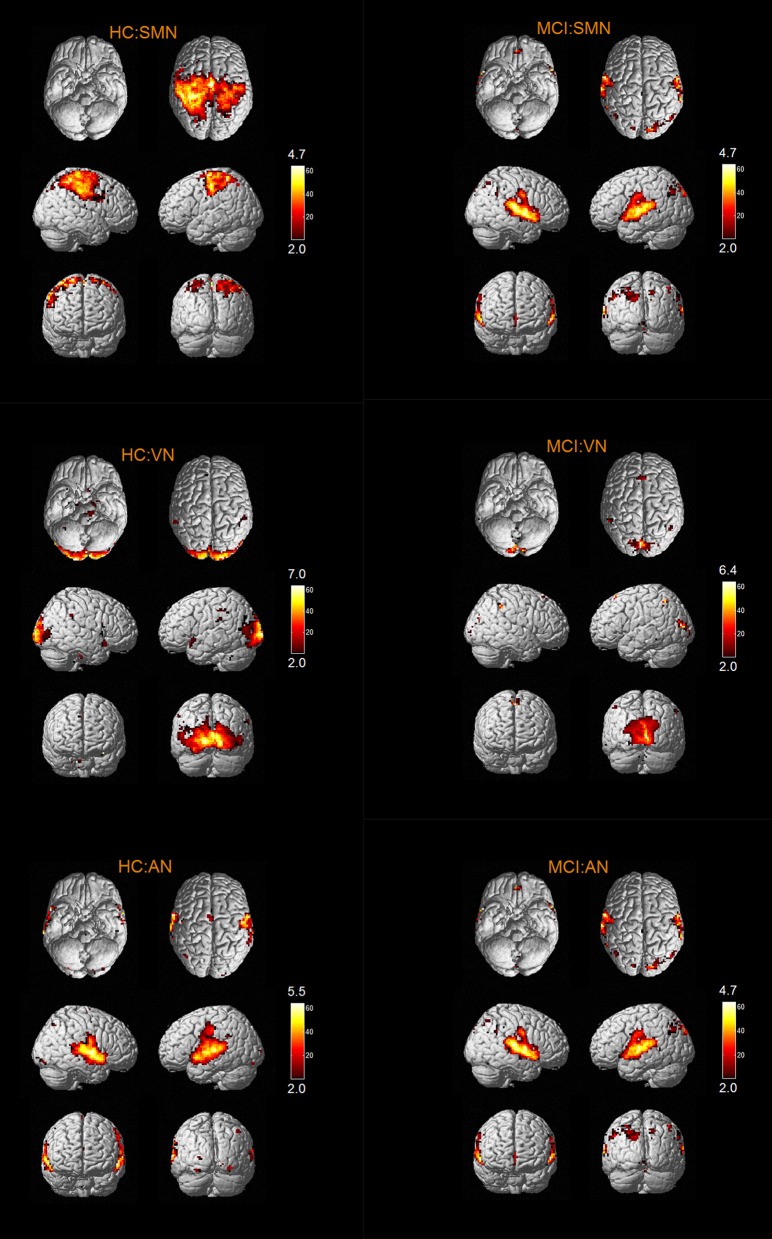
The spatial distribution of VN, AN, DAN in MCI group and HC group.

**Figure 3:**
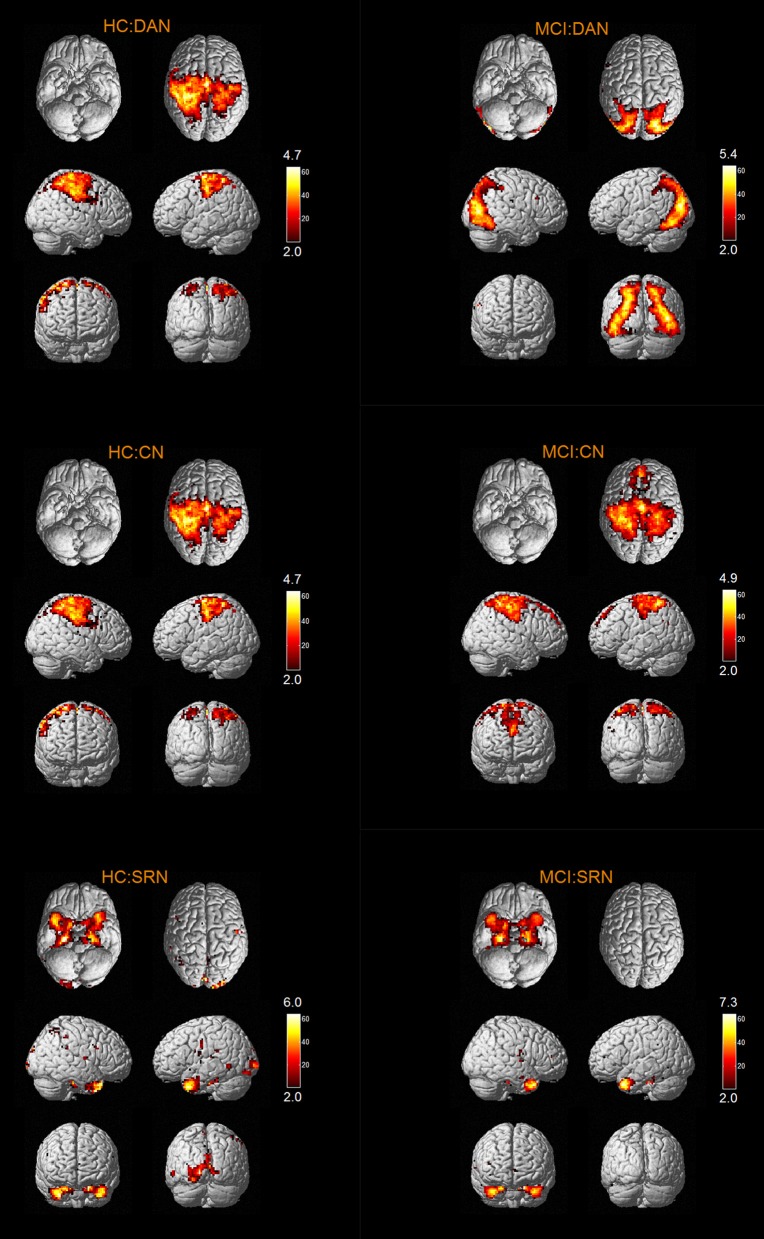
The spatial distribution of CN, SRN in MCI group and HC group.

The results of one-sample *t*-tests (*T* > 2) showed that RSNs of subjects in two groups have typical spatial distribution patterns. The spatial distribution patterns of DMN, CEN, SMN, VN, AN, DAN, CN, and SRN in HC group and MCI group are shown in Figures 1–3.

### Abnormal RSNs in MCI patients

**Figure 4:**
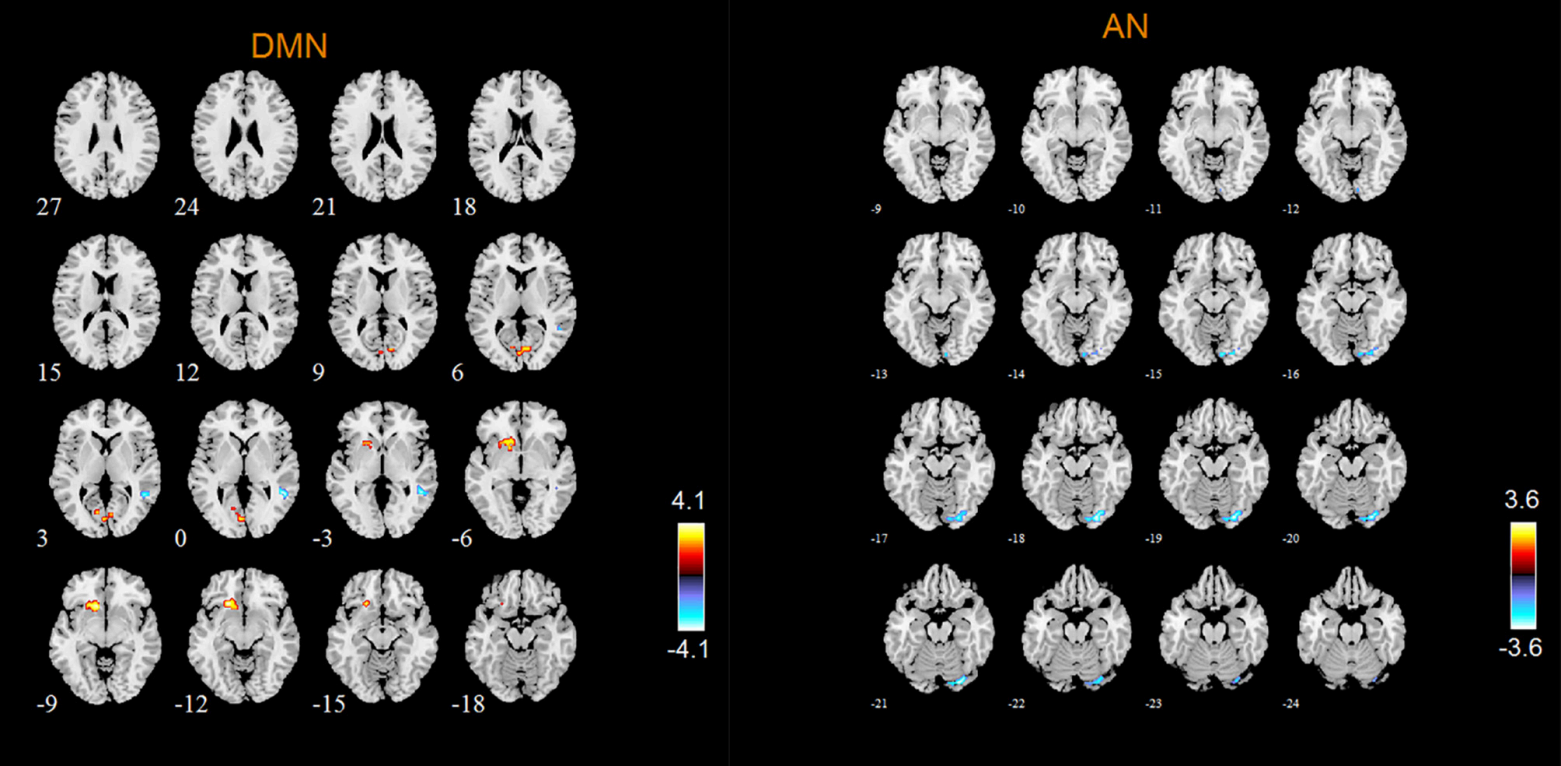
Abnormal brain regions in the DMN, AN, SMN.

**Figure 5:**
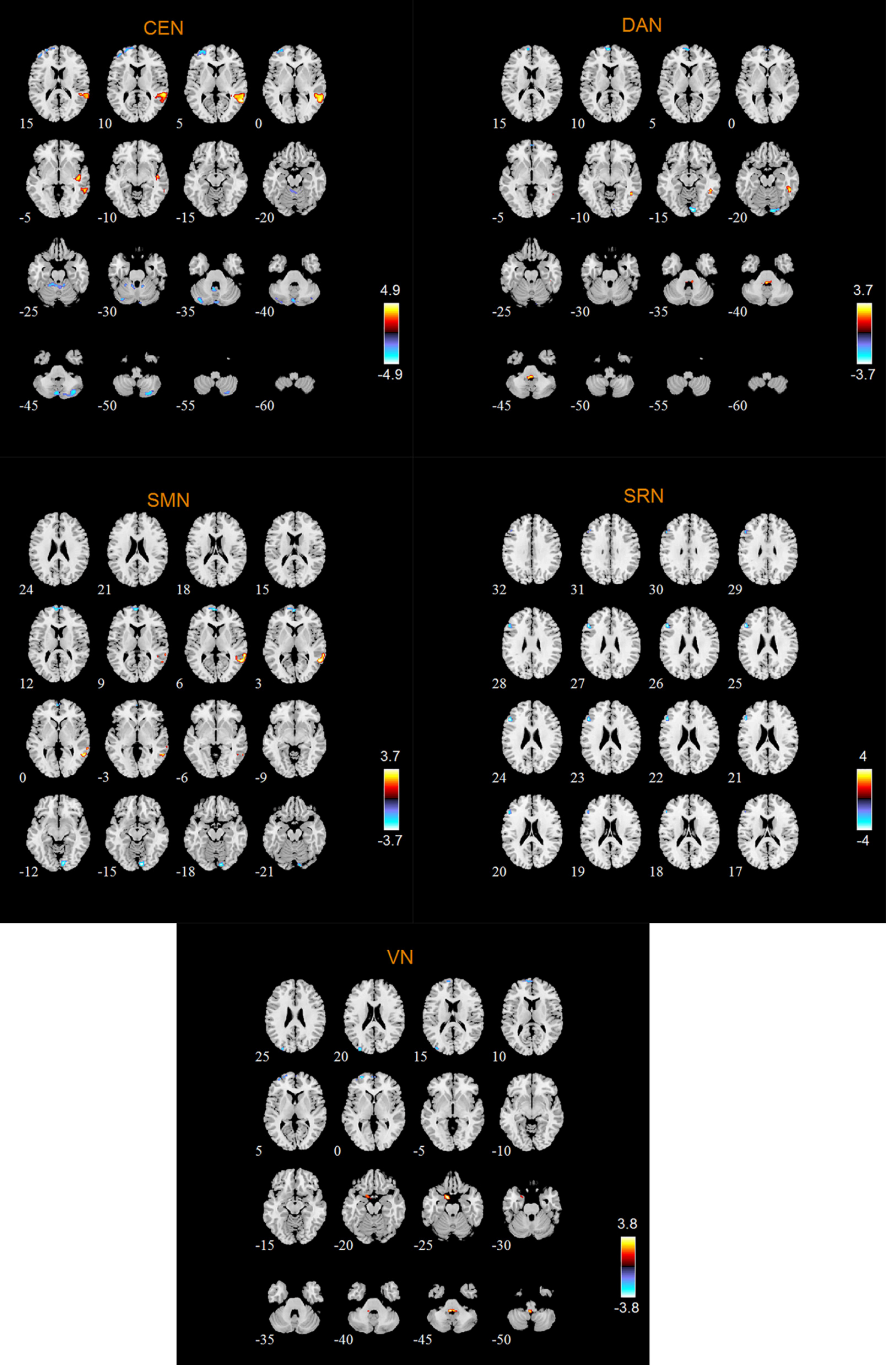
Abnormal brain regions in VN.

According to the two-sample *t*-tests results. we found out abnormal RSNs and brain regions in MCI patients compared to HC as shown in Figures 4, 5. The RSNs with the decreased FC (*P* < 0.05, AlphaSim corrected) in MCI patients included AN and SRN. The RSNs with the increased and decreased FC (*P* < 0.05, AlphaSim corrected) in MCI patients included DMN, CEN, DAN, SMN, VN. The CN did not reveal significant group difference.

Specifically, Table 2 shows the clusters with significant differences of the FC in RSNs of MCI patients. Compared with HC, the abnormal FC is the increased or decreased FC in MCI patients. The abnormal brain regions of the FC in DMN include right middle temporal gyrus (MTG), left orbital part of inferior frontal gyrus (ORBinf), bilateral calcarine fissure and surrounding cortex (CAL), left lingual gyrus (LING).

**Table 2:** Clusters with significant differences of the functional connectivity in RSNs of MCI.

**Cluster**	**Abnormal brain regions**	**The number of voxel**	**Peak coordinates**
**DMN**			
Cluster1	ORBinf.L	78	[−21 24 −12]
Cluster2	MTG.L	36	[51 −48 0]
Cluster3	CAL.L CAL.R LING.L	43	[−3 −81 0]
**AN**			
Cluster1	LING.R	52	[27 −90 −21]
**CEN**			
Cluster1	STG.R	43	[48 −15 −6]
Cluster2	MTG.RSTG.R	250	[60–543]
Cluster3	MFG.L SFGdor.L	101	[−33 606]
**DAN**			
Cluster1	ITG.R	39	[51 −42 −21]
Cluster2	SFGmed.	44	[−663 12]
**SMN**			
Cluster1	MTG.R	67	[60 −54 3]
Cluster2	SFGmed.L	44	[−3 69 6]
**SRN**			
Cluster1	IFGtriang.L	32	[−5127 24]
**VN**			
Cluster1	PHG.L	36	[12 −90 −15]
Cluster2	SFGdor.L SFGmed.L MFG.L	48	[27630]

The abnormal brain regions of the FC in AN include right LING. The abnormal brain regions of the FC in SRN include left triangular part of inferior frontal gyrus (IFGtriang).

The abnormal brain regions of the FC in CEN include left middle frontal gyrus (MFG), left dorsolateral of superior frontal gyrus (SFGdor), right superior temporal gyrus (STG), right MTG.

The abnormal brain regions of the FC in DAN include left medial of superior frontal gyrus (SFGmed), right inferior temporal gyrus (ITG).

The abnormal brain regions of the FC in SMN include left SFGmed, right MTG. The abnormal brain regions of the FC in VN include left SFGdor, left SFGmed, left MFG, left parahippocampal gyrus (PHG).

## Discussion

In this paper, we studied fMRI data of MCI and HC groups using post-nonlinear ICA. We discussed the difference of eight RSNs between MCI and HC groups. Non-linear ICA is a complementary method to linear ICA (Wei et al., 2017a). The decomposition of mixed components by using post-nonlinear ICA is more consistent with the decomposition of actual brain activity, and this method could provide more correct guidance for clinical treatment. Our results showed that some RSNs in MCI patients had abnormality compared to HC. Specifically, the RSNs with the decreased FC in MCI patients included AN and SRN, and the increased and decreased FC in MCI patients included DMN, CEN, DAN, SMN, VN. The CN did not reveal significant group difference.

DMN is considered to be closely related to human episodic memory and self-projection (Liao et al., 2010a). In this study, the abnormal FC of DMN in MCI patients is reflected not only in the increased FC in left ORBinf and left LING, bilateral CAL, but also in the decreased FC in right MTG. Vandenbulcke et al. (2007) studied the activation of brain regions when MCI patients performed the task of reading and image naming, and found that the activation of brain regions in MTG was abnormal, which is the cause of the impairment of word recognition function in MCI patients (Vandenbulcke et al., 2007). Our findings revealed the decreased FC in MTG, which was linked to the impairment of word recognition function in MCI patients. Our research is consistent with previous studies. Other abnormal brain regions of the FC in DMN include ORBinf, LING and CAL. First of all, existing theoretical studies found that ORBinf and orbital part of middle frontal gyrus (ORBmid) of AD patients were severely damaged, which could be observed directly by the anatomy of brain (Van Hoesen et al., 2000; Kumar et al., 2015; Tycko, 2016). Researches have shown that human olfaction is closely related to orbital frontal gyrus, and the decreased olfactory function is one of the clinical manifestations of AD (Wesson et al., 2010; Zou et al., 2016). ORBinf belongs to orbital frontal gyrus. Our results also showed the decreased FC of ORBinf in MCI patients. It is further demonstrated that orbital frontal gyrus is very useful in the process of detecting the transformation from normal aging populations to MCI or AD. In addition, LING is mainly responsible for visual processing (Yang et al., 2015). Li Y et al. (2016) found that the abnormality of LING function was related to the impairment of working memory in MCI patients by using seed correlation analysis method (Li Y et al., 2016). The results of our study also indicated that the FC of LING was abnormal in MCI patients. The last abnormal brain region in DMN is CAL. However, the relationship between CAL and the symptoms of MCI patients is unclear and needs further study.

CEN is related to human cognitive control (Liao et al., 2010a). According to our results, the abnormal FC of CEN in MCI patients is reflected not only in the decreased FC in left MFG and left SFGdor, but also in the increased FC in right STG and right MTG. Döhnel et al. (2008) allowed MCI patients to watch pictures with neutral, positive and negative content, and the process was recorded by fMRI. It was found that the MCI group had a better memory of negative pictures (Döhnel et al., 2008), which indicated that the episodic memory in MCI patients was abnormal. Furthermore, previous studies found that frontal lobe was associated with the episodic memory in human (Pochon et al., 2001). SFGdor belongs to frontal lobe, and our studies found out the decreased FC of SFGdor in MCI patients, which provides a possible explanation for the memory impairment in MCI patients. Another two abnormal brain regions in CEN are right STG and MTG. The temporal lobe is divided into STG, MTG and inferior temporal gyrus (ITG). Specifically, the medial temporal lobe is useful for storing the recent memory in human, and other cortical regions are useful for storing the long-term memory (Gordon and Devinsky, 2003; Lavasani et al., 2016). In our paper, we found that the FC of right STG and MTG was abnormal, which was associated with impaired memory in MCI patients. The last abnormal brain region is left MFG. Wee et al. (2012) found that MFG was primarily responsible for coordinating different information by using seed correlation analysis method (Wee et al., 2012). We found out the decreased FC of left MFG, and this was related to abnormal cognitive control in MCI patients.

DAN is considered as primarily responsible for mediating goal-directed top-down processing (Liao et al., 2010a). According to our results, the abnormal brain regions of the FC in DAN include SFGmed and ITG. The first abnormal brain region of DAN is ITG. Johnson et al. (2006) found that the activation of right ITG was abnormal in MCI patients compared with HC (Johnson et al., 2006). Risacher et al. (2009) also found that the cortex thickness in ITG of the MCI patients decreased in the study of brain structure (Risacher et al., 2009). In our study, we found that the FC of right ITG in MCI patients was abnormal, which is in line with previous studies. Therefore, it is concluded that ITG has a structural and functional change in MCI patients, which can be used as an effective indicator of the clinical diagnosis to predict and monitor the disease. Another abnormal brain region of DAN is SFGmed. The abnormal FC of SFGmed was associated with cognitive impairment in MCI patients.

According to our results, the abnormal FC of VN in MCI patients is reflected not only in the decreased FC in left SFGdor, left SFGmed, left MFG, but also in the increased FC in left PHG. The frontal lobe locates in the front of human brain. It covers about one-third of cerebral hemisphere and includes most of all dopamine sensitive neurons. Dopamine is an important neurotransmitter in the brain, which is closely related to reward mechanism, attention, short-term memory, planning and dopamine systems (Beleza and Pinho, 2011). The executive functions of the frontal lobe include cognitive activity, emotional activity, the ability to predict future results from current behavior, the ability to choose good or bad behavior, the ability to determine the similarities and differences between objects or events (Watanabe et al., 2015). Meanwhile, the frontal lobe has also played an important role in maintaining the long-term memory of human beings in resting-state (Neulinger et al., 2015). SFGdor, SFGmed, MFG belong to frontal lobe. In this study, we found that left SFGdor, left SFGmed and left MFG were abnormal in MCI patients, and these abnormal regions were associated with the memory impairment and attention deficit in MCI patients. Another abnormal brain region in VN is PHG. Celone et al. (2006) used linear ICA found that the hippocampus played a pivotal role not only in the cognitive processing but also in the process of memory retrieval (Celone et al., 2006). Our results showed that the FC of PHG was abnormal, which was related to the decrease of memory and attention disorder in MCI patients.

According to our results, the abnormal FC of AN in MCI patients is reflected in the decreased FC in right LING, and no FC of the brain regions increases. In general, LING is mainly responsible for visual processing. Some researches showed that the abnormality of LING function in MCI patients was associated with impaired working memory in MCI patients (Migo et al., 2014; Kirova et al., 2015). The results of our study also showed that the FC of LING was abnormal, which is in line with previous researches.

According to our results, the abnormal FC of SMN in MCI patients is reflected not only in the decreased FC in left SFGmed, but also in the increased FC in right MTG. As mentioned above, the abnormality of SFGmed in MCI patients was related to the memory impairment and attention deficit, and the abnormal MTG in MCI patients was associated with memory impairment.

According to our results, the abnormal FC of SRN in MCI patients is reflected in the decreased FC in left IFGtriang, and no FC of the brain regions increases. As the FC of IFGtriang in MCI patient was abnormal, it is suggested that the patient's ability to maintain a long-term memory is impaired (Miotto et al., 2014; Lin et al., 2016).

In the process of detecting the abnormal FC and lesions in MCI patients, the results of our approach are not only consistent with the results obtained by the traditional methods, such as seed correlation analysis method, linear ICA, global functional connectivity method, but also some new abnormal FC and lesions in MCI patients are obtained by our method. The abnormalities of the FC in PHG, MFG, and LING were found out by our method which is in accordance with the traditional methods. We have also found the abnormal FC in SFGdor, SFGmed, CAL, which is not found out by traditional methods. The abnormal FC in these brain regions are in agreement with the clinical symptoms of MCI patients. These findings further provide strong evidence for the correctness of our results.

Our study contributes to introducing the post-nonlinear ICA method to analyze fMRI data, but it also has some limitations. First of all, our non-linear ICA method does not provide the information of the FC of limbic system, which needs to be explored in future research. Secondly, although a large number of studies consistently demonstrated that spontaneous brain activity could be organized into RSNs (Liao et al., 2010b), a complete description of the brain functional architecture has not yet been provided by the RSNs documents at present and the neurophysiological meaning of RSNs is still unclear. In addition, we have not studied the correlation between fMRI data and clinical scoring in MCI patients, which is needed to be explored in future studies.

## Ethics statement

This study was carried out in accordance with the recommendations of Health Insurance Portability and Accountability Act (HIPAA) guidelines, National Institutes of Health (NIH) Combined Neuroscience Institutional Review Board with written informed consent from all subjects. All subjects gave written informed consent in accordance with the Declaration of Helsinki. The protocol was approved by the National Institutes of Health (NIH) Combined Neuroscience Institutional Review Board.

## Author contributions

XB proposed the design of the work and revised it critically for important intellectual content. LW and QS carried out the experiment for the work and drafted part of the work. JZ and QX collected, interpreted the data and drafted part of the work. All the authors approved the final version to be published and agreed to be accountable for all aspects of the work in ensuring that questions related to the accuracy or integrity of any part of the work are appropriately investigated and resolved.

### Conflict of interest statement

The authors declare that the research was conducted in the absence of any commercial or financial relationships that could be construed as a potential conflict of interest.

## Footnotes

^1^Detials of this method are introduced in the Appendix.
