# Supplementary material for: Non-linear ICA Analysis of Resting-State fMRI in Mild Cognitive Impairment
Source: Front Neurosci. 2018 Jun 19;12:413. doi: 10.3389/fnins.2018.00413 (PMC6018085; doi:10.3389/fnins.2018.00413)
Supplement: Supplementary file 1 [file Data_Sheet_1.docx]

Appendix

**Non-linear ICA and its application in fMRI**

As the brain is a very complex system ([Poldrack and Farah 2015](#_ENREF_1)) and the mixture of ICs is not necessarily linear ([Wu, Meng et al. 2017b](#_ENREF_2)), it is natural to extend the linear model to the non-linear model. In many practical applications, the mixture of signals is often more complex than linear mixing ([Zhang and Wang 2017](#_ENREF_3)), and in some cases, the separation algorithm based on linear mixed assumption used in non-linear mixed problems may lead to complete failure or incorrect results.

**Principle of non**-**linear ICA method**

For a transient mixed signal, its non-linear mixed model can be expressed as the following form:

$\boldsymbol{X}\boldsymbol{=f(}\boldsymbol{S}\boldsymbol{)}$ (1)

In the formula, $\boldsymbol{X=}{\boldsymbol{(}\boldsymbol{x}_{\boldsymbol{1}}\boldsymbol{,}\boldsymbol{x}_{\boldsymbol{2}}\boldsymbol{,\cdots,}\boldsymbol{x}_{\boldsymbol{n}}\boldsymbol{)}}^{\boldsymbol{T}}$ is the observation signal, and its dimension is $n$. $\boldsymbol{f}$ is an unknown numerical mixing function. $\boldsymbol{S=}{\boldsymbol{(}\boldsymbol{s}_{\boldsymbol{1}}\boldsymbol{,}\boldsymbol{s}_{\boldsymbol{2}}\boldsymbol{\cdots,}\boldsymbol{s}_{\boldsymbol{m}}\boldsymbol{)}}^{\boldsymbol{T}}$ is the unknown source signal of $m$-dimensional, and its elements are $m$ unknown ICs. In generally, we assume that the number of ICs equals to the number of mixed signals, that is, $m=n$. Then the principle of non-linear ICA is to find a mapping $g:R^{n}\to R^{n}$ through observed signal $\boldsymbol{X}$ and source signal $\boldsymbol{S}$**,** where $\boldsymbol{S}$ satisfies with the statistical independence hypothesis. The source signal $\boldsymbol{S}$ could be estimated by observation signal $\boldsymbol{X}$ and the mapping $\boldsymbol{g}$.

It is assumed that the output signal of the mixed signal separation system is:

$$\boldsymbol{Y=}{\boldsymbol{(}\boldsymbol{y}_{\boldsymbol{1}}\boldsymbol{,}\boldsymbol{y}_{\boldsymbol{2}}\boldsymbol{,\cdots,}\boldsymbol{y}_{\boldsymbol{n}}\boldsymbol{)}}^{\boldsymbol{T}}\boldsymbol{\in}\boldsymbol{R}^{\boldsymbol{n}}$$

Then $\boldsymbol{Y}$ is the estimate of $\boldsymbol{S}$, and the mathematical model is represented as:

$\boldsymbol{Y=g}\left( \boldsymbol{X} \right)\boldsymbol{=}\hat{\boldsymbol{S}}$ (2)

The general non-linear ICA mixed separation process is shown in Figure 6:

$$\boldsymbol{y}_{\boldsymbol{n}}$$

$$\boldsymbol{y}_{\boldsymbol{2}}$$

$$\boldsymbol{x}_{\boldsymbol{n}}$$

$$\boldsymbol{s}_{\boldsymbol{n}}$$

$$\boldsymbol{s}_{\boldsymbol{2}}$$

$$\boldsymbol{y}_{\boldsymbol{1}}$$

$$\boldsymbol{x}_{\boldsymbol{1}}$$

$$\boldsymbol{s}_{\boldsymbol{1}}$$

$$\vdots$$

$$\vdots$$

$$\boldsymbol{x}_{\boldsymbol{2}}$$

***g***

***f***

$$\vdots$$

Figure 6 The schematic diagram of non-linear independent component analysis

**Application of post**-**nonlinear ICA in fMRI**

For a general non-linear mixed model, an important and special case is the so-called post-nonlinear mixed model. Specifically, a signal firstly goes through a linear channel, and then the non-linear characteristics is introduced. Thus, this special non-linear mixed model is also in line with the actual application scenario, and could be represented by the following formula:

$x_{i}=f_{i}(\sum_{j=1}^{n} a_{ij}s_{j})$ $i=1,\cdots,n$ (3)

where the source signal $s_{j} (i=1,\cdots,n)$ firstly goes through the simple ICA linear mixture, and then through the non-linear function $f_{i}$ to form the final observation signal $x_{i}$. Then the matrix form of equation (3) could be obtained as:

$\boldsymbol{X=f(AS)}$ (4)

In the following, the application of the post-nonlinear mixed model to fMRI is discussed in detail. It is assumed that fMRI image sequences include $k$images, and each image has $n$ voxels, and the i-th image is denoted by $k_{i}$. The process of separating independent components by nonlinear ICA is as follows:

First, $\left[ s_{1} , s_{2} ,\ldots, s_{n} \right]^{T}$represents independent components, and $\left[ c_{1} , c_{2} ,\ldots, c_{n} \right]^{T}$ is a linear combination of independent components $\left[ s_{1} , s_{2} ,\ldots, s_{n} \right]^{T}$, which could be regarded as the simple linear ICA. Thus we could get:

$\boldsymbol{C=AS}$ (5)

The equation (5) can also be expressed as the following formula:

$\left[ \begin{aligned} c_{1} \\ \vdots\\ c_{n} \end{aligned} \right]=\left[ \begin{matrix} a_{11} & \cdots& a_{1n} \\ \vdots& \vdots& \vdots\\ a_{n1} & \cdots& a_{nn} \end{matrix} \right]\left[ \begin{aligned} s_{1} \\ \vdots\\ s_{n} \end{aligned} \right]$

where

$\boldsymbol{A}=\left[ \begin{matrix} a_{11} & \cdots& a_{1n} \\ \vdots& \vdots& \vdots\\ a_{n1} & \cdots& a_{nn} \end{matrix} \right]$,

that is, the independent component $\left[ s_{1} , s_{2} ,\ldots, s_{n} \right]^{T}$is linearly mixed into $\boldsymbol{C}$ by matrix $\boldsymbol{A}$**.**

We also assumed that$k_{1}=\left[ x_{11} x_{12} ,\ldots, x_{1n} \right]$, where$k_{1}$ indicates the intensity of $m$voxels at a certain point in time. Then we could acquire

$\left[ \begin{aligned} k_{1} \\ \vdots\\ k_{n} \end{aligned} \right]=\left[ \begin{matrix} x_{11} & \ldots& x_{1n} \\ \vdots& \vdots& \vdots\\ x_{n1} & \cdots& x_{nn} \end{matrix} \right]$ (6)

where$\left[ k_{1} , k_{2} ,\ldots, k_{n} \right]^{T}$ stands for the intensity of the voxel at $n$ time point. In this paper, we could obtain the mixed signals $\left[ k_{1} , k_{2} ,\ldots, k_{n} \right]^{T}$ by the linear combinations of independent components$\left[ c_{1} , c_{2} ,\ldots, c_{n} \right]^{T}$ and the non-linear function $f$.

$\left[ \begin{aligned} k_{1} \\ \vdots\\ k_{n} \end{aligned} \right]=f(\left[ \begin{aligned} c_{1} \\ \vdots\\ c_{n} \end{aligned} \right])$ (7)

The above process is the mixing process, and the following is the separation process.

The separation process of nonlinear mixed components can be transformed to the process of finding the non-linear mapping $g$to make $Y$ equal to$g(k)$, that is,

$\left[ \begin{aligned} Y_{1} \\ \vdots\\ Y_{n} \end{aligned} \right]=g(\left[ \begin{aligned} k_{1} \\ \vdots\\ k_{n} \end{aligned} \right])$ (8)

where

$\left[ \begin{aligned} k_{1} \\ \vdots\\ k_{n} \end{aligned} \right]$=$\left[ \begin{matrix} x_{11} & \ldots& x_{1n} \\ \vdots& \vdots& \vdots\\ x_{n1} & \cdots& x_{nn} \end{matrix} \right]$, $\left[ k_{1} , k_{2} ,\ldots, k_{n} \right]^{T}$ is the mixed signal we observed, and $\left[ Y_{1} , Y_{2} ,\ldots, Y_{n} \right]^{T}$ is the estimate of the source signal $\boldsymbol{S}$.

The schematic diagram of the post-nonlinear ICA is shown in Figure 7

W

A

$$\boldsymbol{y}_{\boldsymbol{1}}$$

$$\boldsymbol{s}_{\boldsymbol{1}}$$

$$\boldsymbol{x}_{\boldsymbol{1}}$$

$$\boldsymbol{y}_{\boldsymbol{n}}$$

$$\boldsymbol{y}_{\boldsymbol{2}}$$

$$\boldsymbol{s}_{\boldsymbol{n}}$$

$$\boldsymbol{s}_{\boldsymbol{2}}$$

$$\boldsymbol{x}_{\boldsymbol{n}}$$

$$\boldsymbol{x}_{\boldsymbol{2}}$$

$$\boldsymbol{g}_{\boldsymbol{n}}$$

$$\boldsymbol{g}_{\boldsymbol{2}}$$

$$\boldsymbol{g}_{\boldsymbol{1}}$$

$$\boldsymbol{f}_{\boldsymbol{n}}$$

$$\boldsymbol{f}_{\boldsymbol{2}}$$

$$\boldsymbol{f}_{\boldsymbol{1}}$$

$$\vdots$$

$$\vdots$$

$$\vdots$$

$$\vdots$$

Non-linear correction

Non-linear distortion

Linear mixed

Linear separation

Separation System

Mixed System

Figure 7 The schematic diagram of post-nonlinear independent component annlysis

In Figure 7, the left part is a mixed system of the post-nonlinear model, and the right part is the corresponding separation system. The separation signal $\boldsymbol{Y}$ is shown in the following formula:

$y_{i}=\sum_{j=1}^{n} w_{ij}g_{j}(x_{j})$ (9)

For linear ICA:

$\boldsymbol{X=AS}$

$$\boldsymbol{Y=WX}$$

where $\boldsymbol{S}$ is a blind source, $\boldsymbol{A}$ is a mixed matrix, $\boldsymbol{W}$ is the demixing matrix, $\boldsymbol{X}$ is the observed signal, and $\boldsymbol{Y}$ is the estimate of $\boldsymbol{S}$.

For post-nonlinear ICA:

$\boldsymbol{X}=f\left( \boldsymbol{AS} \right)$

$$\boldsymbol{Y}=\boldsymbol{W}g(\boldsymbol{x})$$

where $\mathbf{X=}\sqrt[\mathbf{3}]{\mathbf{AS}}$.

In order to get the blind source, we solving process is as follows:

$$\boldsymbol{X=}\sqrt[\boldsymbol{3}]{\boldsymbol{AS}}$$

$$\boldsymbol{X}^{\boldsymbol{3}}\boldsymbol{=AS}$$

$$\boldsymbol{A}^{\boldsymbol{-1}}\boldsymbol{X}^{\boldsymbol{3}}\boldsymbol{=}\boldsymbol{A}^{\boldsymbol{-1}}\boldsymbol{AS}$$

Then we finally get

$$\boldsymbol{W}\boldsymbol{X}^{\boldsymbol{3}}\boldsymbol{=Y}$$

where the demixing matrix $\boldsymbol{W}$ is $\boldsymbol{A}^{\boldsymbol{-1}}$, $g(\boldsymbol{x})$ is $\boldsymbol{X}^{\boldsymbol{3}}$and $\boldsymbol{Y}$ is the estimate of $\boldsymbol{S}$. It is noted that we let$g(\boldsymbol{x})$ equal to $\boldsymbol{X}^{\boldsymbol{3}}$, because too high order leads to over-fitting and too low order leads to under-fitting.

Poldrack, R. A. and M. J. Farah (2015). "Progress and challenges in probing the human brain." Nature **526**(7573): 371-379.

Wu, Y., et al. (2017b). "An input-based triggering approach to leader-following problems." Automatica **75**: 221-228.

Zhang, H. and J. Wang (2017). "Active Steering Actuator Fault Detection for An Automatically-steered Electric Ground Vehicle." IEEE Transactions on Vehicular Technology **PP**(99): 1-1.
